# Elongation Factor 1 alpha interacts with phospho-Akt in breast cancer cells and regulates their proliferation, survival and motility

**DOI:** 10.1186/1476-4598-8-58

**Published:** 2009-08-03

**Authors:** Luisa Pecorari, Oriano Marin, Chiara Silvestri, Olivia Candini, Elena Rossi, Clara Guerzoni, Sara Cattelani, Samanta A Mariani, Francesca Corradini, Giovanna Ferrari-Amorotti, Laura Cortesi, Rita Bussolari, Giuseppe Raschellà, Massimo R Federico, Bruno Calabretta

**Affiliations:** 1 Department of Biomedical Sciences, Section of General Pathology, University of Modena and Reggio Emilia, 41100 Modena, Italy; 2 CRIBI Biotechnology Center, Department of Biological Chemistry, University of Padova, 35122 Padova, Italy; 3 Department of Oncology and Haematology, University of Modena and Reggio Emilia, 41100 Modena, Italy; 4 ENEA Research Center Casaccia, Biotechnology Unit, Section of Toxicology and Biomedical Sciences, 00123 S. Maria di Galeria, Roma, Italy; 5 Kimmel Cancer Center, Thomas Jefferson University, Philadelphia, PA, 19197 USA

## Abstract

**Background:**

Akt/PKB is a serine/threonine kinase that has attracted much attention because of its central role in regulating cell proliferation, survival, motility and angiogenesis. Activation of Akt in breast cancer portends aggressive tumour behaviour, resistance to hormone-, chemo-, and radiotherapy-induced apoptosis and it is correlated with decreased overall survival. Recent studies have identified novel tumor-specific substrates of Akt that may provide new diagnostic and prognostic markers and serve as therapeutic targets. This study was undertaken to identify pAkt-interacting proteins and to assess their biological roles in breast cancer cells.

**Results:**

We confirmed that one of the pAkt interacting proteins is the Elongation Factor EF1α. EF1α contains a putative Akt phosphorylation site, but is not phosphorylated by pAkt1 or pAkt2, suggesting that it may function as a modulator of pAkt activity. Indeed, downregulation of EF1α expression by siRNAs led to markedly decreased expression of pAkt1 and to less extent of pAkt2 and was associated with reduced proliferation, survival and invasion of HCC1937 cells. Proliferation and survival was further reduced by combining EF1α siRNAs with specific pAkt inhibitors whereas EF1α downregulation slightly attenuated the decreased invasion induced by Akt inhibitors.

**Conclusion:**

We show here that EF1α is a pAkt-interacting protein which regulates pAkt levels. Since EF1α is often overexpressed in breast cancer, the consequences of EF1α increased levels for proliferation, survival and invasion will likely depend on the relative concentration of Akt1 and Akt2.

## Background

Breast cancer is the most common cancer among women in the European Union: each year, 60,000 women die of breast cancer and 150,000 new cases are diagnosed. Proliferation and survival of breast cancer cells are regulated by steroid hormones, growth factors and their receptors through the activation of signal transduction pathways which, in many cases, are aberrantly activated [1]. The phosphatidylinositol-3 kinase (PI-3K) pathway has crucial roles in breast cancer [2], and can be altered at multiple levels, including mutations of the PI-3K catalytic subunit [3] or of its "upstream" or "downstream" regulator/effectors such as PTEN and AKT [4,5]. Akt/PKB is a serine/threonine kinase that has attracted much attention because of its central role in regulating several cellular processes such as proliferation, angiogenesis, motility and survival [6]. Activation of Akt in breast cancer portends aggressive tumour behaviour, resistance to hormone-, chemo-, and radiotherapy-induced apoptosis and it is correlated with decreased overall survival [7]. Recent studies have identified novel tumor-specific substrates of Akt that may provide new diagnostic and prognostic markers and serve as therapeutic targets [8]. In light of the central role of Akt in the regulation of cell survival, specific inhibitors of this kinase might induce apoptosis when used alone or in combination with standard cancer chemotherapeutics. In this regard, suppression of Akt activity by small molecule allosteric inhibitors [9] sensitizes many tumour cell lines to apoptotis induced by DNA damage, microtubule-binding agents, targeted therapeutics and ionizing radiation [10] suggesting that Akt inhibitors may enhance the efficacy of chemotherapy and radiotherapy in breast cancer. However, the use of Akt inhibitors in anti-cancer therapies should take into account that neoplastic cells express variable levels of Akt isoforms that may have different functions, including the distinct ability of pAkt1 or pAkt2 to regulate migration and invasion of breast cancer cells [11,12].

This study was undertaken to identify additional pAkt-interacting proteins and to assess their biological roles in breast cancer cells. To this end, we used anti-pAkt immunoprecipitation followed by mass spectrometry of pAkt-associated proteins; of several interacting proteins containing putative Akt phosphorylation sites (RXRXX S/T Ψ), we selected for further studies the eukaryotic Elongation Factor 1 alpha (EF1α).

EF1α consists of two isoforms, EF1α1 and EF1α2, that share >90% sequence identity and have the same function in mRNA translation [13], but markedly different expression patterns [14,15]. Both proteins appear to be involved in tumour development and progression [16] and, relative to normal tissues, expression of EF1α1 and EF1α2 is more abundant in breast cancer samples [17]. Since EF1α1 is expressed at high levels in normal breast tissues while EF1α2 is barely detectable, overexpression of EF1α2 is more likely be biologically relevant in breast cancer. Recent studies have also correlated the expression of EF1α2 with ER/HER-2 expression, lymph node status, survival, tumor size and p53 mutations [17,18].

In this study, we investigated the functional relationship between EF1α and Akt and the biological consequences of downregulating EF1α expression for proliferation, survival and invasion of breast cancer cells. We show here that EF1α binds only to pAkt but is not an in vitro substrate of active Akt1 or Akt2. Downregulation of EF1α expression led to decreased expression of pAkt1 and to less extent of pAkt2, inhibited proliferation, colony formation and invasion and promoted apoptosis of HCC1937 cells. The combination of EF1α siRNA with pAkt inhibitors further reduced proliferation, survival and clonogenic potential of HCC1937 cells, suggesting that these effects are, in part, Akt-independent; downregulation of EF1α expression attenuated the inhibition of cell motility induced by Akt inhibitors, consistent with the possibility that complete inhibition of pro-metastatic Akt1 activity by EF1α RNAi promotes a slight increase in the invasiveness of HCC1937 cells. Together, these observations suggest that EF1α is a regulator of Akt activity and that the biological consequences of modulating EF1α expression in breast cancer cells reflect Akt-dependent and -independent mechanisms.

## Results and discussion

### Identification of phospho-Akt interacting proteins

To identify novel pAkt- interacting proteins in breast cancer cells, proteins associated with pAkt were analyzed by mass spectrometry. To this end, whole lysates from HCC1937 or SkBr3 breast cancer cells were immunoprecipitated with an anti-pAkt antibody and the pAkt interactome was compared to that from anti-Akt1/Akt2 immunoprecipitates (IPs) of pAkt-depleted lysates (data not shown). Proteins interacting with pAkt in SkBr3 cells were also identified by comparing the anti-Akt IPs of untreated cells and of cells treated with the dual pAkt1/pAkt2 (pAkt1/2) Merck's inhibitor [9]. The anti-Akt IPs were then subjected to 2-D electrophoresis and visualized by silver staining (data not shown). Together, these two procedures allowed us to identify several proteins preferentially or exclusively present in the pAkt interactomes.

### EF1α interacts with phospho-Akt

Spots predominantly present in the anti-pAkt IPs were excised from the gels and subjected to tryptic digestion for mass spectrometry identification. One of the p-Akt interacting protein identified in the HCC1937 and SkBr3 cells is EF1α, previously shown to activate Akt [19].

**Figure 1:**
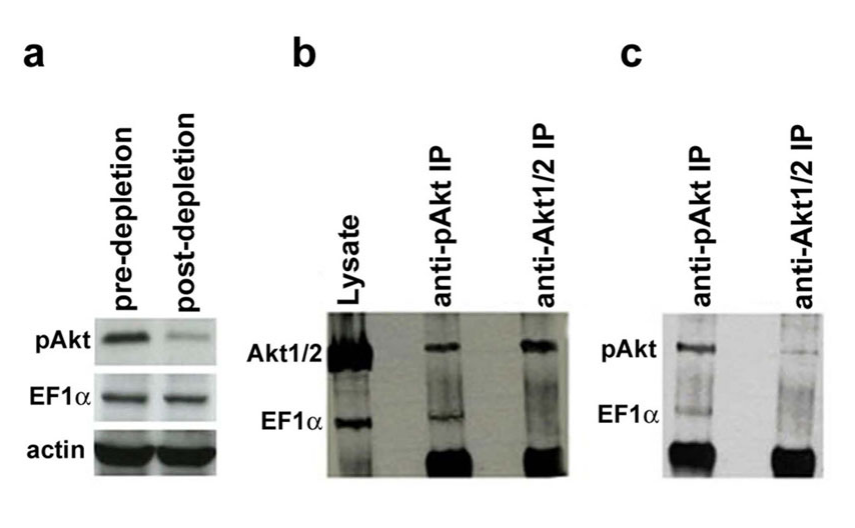
**Interaction between EF1α and Akt in SkBr3 cells**. SkBr3 whole cell lysates and lysates of SkBr3 cells subjected to anti-pAkt (Ser473) IP in saturating conditions to deplete the active kinase were immunoprecipitated with anti-pAkt or anti-Akt1/Akt2 antibody, respectively. IPs were separated in two equal amounts and subjected to SDS-PAGE and western blotting using anti-Akt1/2 (panel b) and anti-pAkt (panel c) antibodies to distinguish pAkt and unphosphorylated Akt and to avoid superimposition of adjacent bands and background interference. Levels of associated EF1α were also detected in both membranes. Western blots show: (a) expression of pAkt and EF1α in whole cell and p-Akt-depleted lysates. Expression of β-Actin was used as loading control; expression of total Akt (Akt1/Akt2) and EF1α (b) or of pAkt and EF1α (c) in the anti-pAkt and in the anti-Akt (Akt1/Akt2) IPs from p-Akt-depleted SkBr3 cell lysate. In the anti-Akt1/2 IPs obtained after pAkt depletion, levels of residual pAkt (panel c) were negligible.

To confirm the interaction between pAkt and EF1α, anti-pAkt and anti-Akt1/2 IPs from pAkt-depleted SkBr3 cell lysate were subjected to western blotting with an anti-EF1α antibody. EF1α was detected as a 50 kDa band only in the anti-pAkt IPs (Fig. 1b and 1c). Co-immunoprecipitation/Western blot experiments performed in HCC1937 cells showed identical results (see Additional File 1).

To further characterize the specificity of the pAkt-EF1α interaction, additional experiments were performed with antibodies specific for the Akt1 and Akt2 isoforms in lysate of untreated or pAkt inhibitor-treated cells.

**Figure 2:**
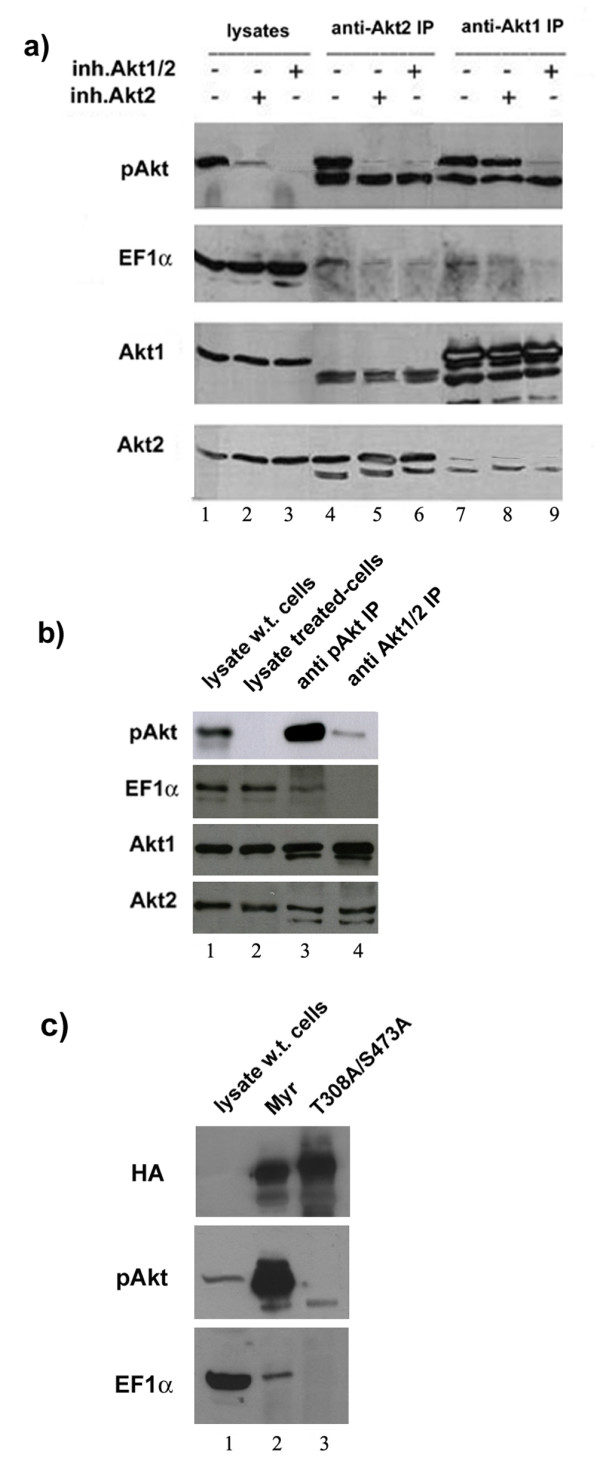
**(a) Western blotting of HCC1937 cell lysates and anti-Akt2 or anti-Akt1 IPs by anti-EF1α, anti-pAkt Ser473, anti-Akt1 and anti-Akt2 antibodies**. Selective inhibitors of pAkt2 and pAkt1/2 were used to reduce the concentration of active pAkt. (b) Western blot shows expression of pAkt, Akt1, Akt2 and EF1α in untreated and pAkt1/2 inhibitor-treated SkBr3 cell lysate (lanes 1 and 2) and in anti-pAkt (from untreated cell lysates) and anti-Akt1/2 IPs (from pAkt inhibitor-treated cells) (lanes 3 and 4). (c) Western blot shows co-immunoprecipitation of HA-tagged pAkt1 and EF1α in SkBr3 cells transfected with HA-tagged Myr-Akt1 (lane 2) or T308A/S473A Akt1 (lane 3) expression vector. Lysate of SkBr3 wild type cells (lane 1) was used as control.

Treatment with the Akt1/2 inhibitor led to complete disappearance of pAkt in the HCC1937 cell lysate (Fig. 2a, lane 3); instead, levels of pAkt in lysate of cells treated with the Akt2 inhibitor were still detectable (Fig. 2a, lane 2), suggesting that they represent residual pAkt1 and that Akt2 is the predominantly phosphorylated isoform in HCC1937 cells. Levels of EF1α, total Akt1 and total Akt2 remained constant after treatment with the inhibitors (Fig. 2a, lanes 1–3). EF1α was detectable in the anti-Akt2 IPs from untreated cells and, at lower levels, in the anti-Akt2 IP from lysate of Akt inhibitor-treated cells (Fig. 2a, lanes 5 and 6) suggesting that low levels of pAkt2 are still present in the anti Akt2 immunocomplex from Akt inhibitor-treated cells.

EF1α was also found in the anti-Akt1 IP (Fig. 2a, lane 7), and formation of the complex appears to depend on Akt1 phosphorylation since the amount of immunoprecipitated EF1α was markedly reduced in cells treated with the Akt1/2 inhibitor (Fig. 2a, lane 9).

The phosphorylation-dependent association of Akt with EF1α was also tested in untreated and in pAkt1/2 inhibitor-treated SkBr3 cells. Also in this case, EF1α was found exclusively in the anti-pAkt IP (Fig. 2b, lane 3).

The association of pAkt-1 and EF1α was further assessed in SkBr3 cells transfected with HA-tagged Myr-Akt1 or the phosphorylation-deficient T308A/S473A Akt1 mutant. Ectopically expressed Myr-Akt1 was abundantly phosphorylated (anti-phospho Ser473 western blotting) and interacted with endogenous EF1α (Fig. 2c, lane 2); by contrast, the double mutant T308A/S473A Akt1 was not phosphorylated and failed to interact with EF1α (Fig. 2c, lane 3).

### In vitro kinase assays in the presence of EF1α

The interaction of EF1α with pAkt and the finding that EF1α contains a putative Akt phosphorylation site (amino acids 67–72) suggested that EF1α might be an Akt substrate. Thus, we synthesized a GST fusion protein (GST-EF1α) that contains amino acids 1–173 of human EF1α and includes the putative Akt phosphorylation site and surrounding amino acids.

**Figure 3:**
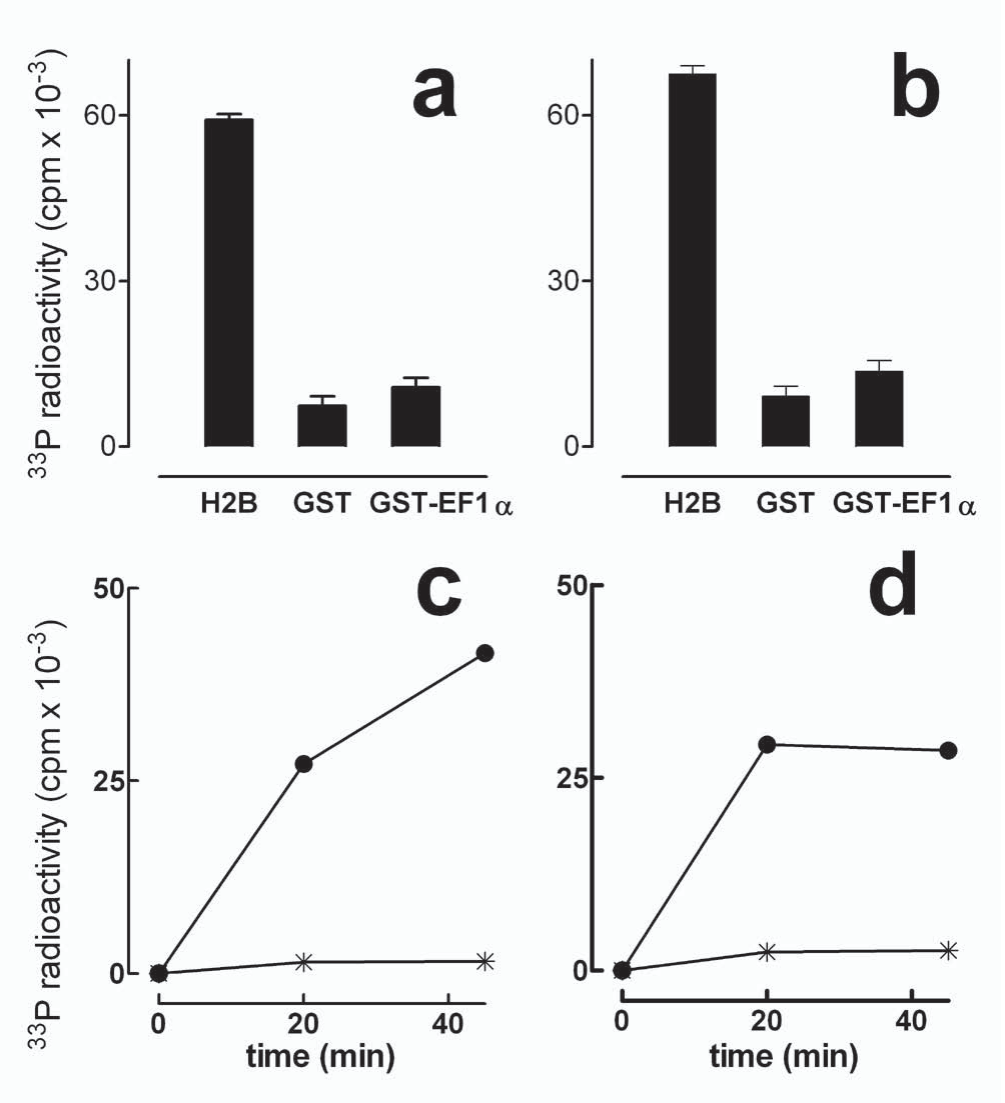
**(a and b): Phosphorylation of GST fusion protein containing amino acids 1–173 of human EF1α by Akt**. Phosphorylation of GST-EF1α by Akt 1 and Akt 2 is reported in panel a and b, respectively, in comparison with histone H2B and GST used as controls. 1.5 μg of each substrate was incubated with 100 ng of Akt isoforms for 45 min in the presence of 50 μM [γ^33^P] ATP (specific activity, 4000 cpm/pmol) as detailed in Materials and Methods. Data are the mean of three independent experiments with SD indicated by vertical bars. (c and d): Phosphorylation of EF1α KAERERGITID peptide (EFtide) by Akt. 100 ng of active Akt 1 (panel c) or 2 (panel d) was incubated for the indicated time with peptide KAERERGITID (EFtide) or Akt substrate peptide RPRAATF (AKTide). Phosphorylation of EFtide (Star) and AKTide (Black dot) was performed at 1 mM and 25 μM concentration, respectively. The amount of ^33^P incorporated was determined as described in the Materials and Methods. Data reported are the mean of three independent experiments.

GST-EF1α, GST alone as negative control and histone H2B as positive control, were used as potential "in vitro" substrates of active Akt isoforms 1 and 2. Phosphorylation, detected by means of phosphocellulose filters (Fig. 3, panels a and b) and SDS/PAGE and autoradiography (see Additional File 2) showed that the GST-EF1α fusion protein is not a pAkt1 (panel a) or pAkt2 (panel b) substrate, whereas histone H2B, used as positive control, was readily phosphorylated.

To further investigate whether EF1α is a *bona fide *substrate, a short peptide that includes the putative Akt phosphorylation site (EFtide) was used as pAkt1 and/or pAkt2 substrate in "in vitro" kinase assays. In these experiments, a specific peptide substrate (AKTide) common to pAkt1 and pAkt2 was used as positive control. In three separate experiments, the EF1α peptide was not phosphorylated while the AKTide peptide was readily phosphorylated (Fig. 3, panel c and d)

### Silencing of EF1α expression suppresses pAkt levels in breast cancer HCC1937 cells

**Figure 4:**
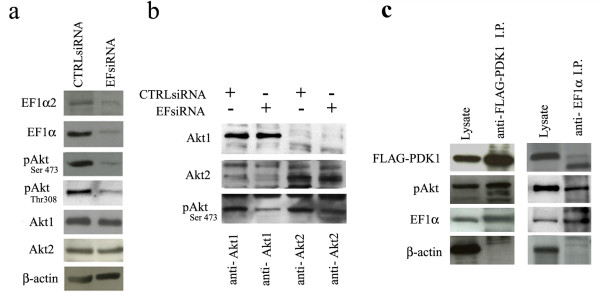
**Effect of EF1α expression on pAkt, Akt1 and Akt2 levels**. a) Western blot shows levels of the indicated proteins (including β-actin for normalization) in HCC1937 cells 24 h after transfection with CTRLsiRNA or EF1α siRNAs; b) Western blot shows expression of pAkt in anti-Akt1 or anti-Akt2 IPs of CTRL or EF1α siRNA-transfected HCC1937 cells; c) Western blot shows pAkt and EF1α expression in lysate or IPs (anti-FLAG or anti-EF1α) of SkBr3 cells transfected with FLAG-PDK1.

To investigate the function of EF1α in breast cancer cells, EF1α expression was silenced by RNA interference using pre-determined conditions (see Additional File 3). 24 h after transfection with the EFsiRNA, cells were lysed and extracts analysed by SDS-PAGE and western blotting with specific antibodies to test the effects of EF1α downregulation on expression and activation of Akt proteins (Fig. 4a).

Since the siRNA used for RNA-interference does not distinguish between EF1α1 and EF1α2, western blots were performed with an antibody detecting both isoforms or with an antibody specific for EF1α; both confirmed that total EF1α levels and expression of EF1α2 were downregulated in HCC1937 cells treated for 24 h with the EF1α siRNA (Fig. 4a). Total levels of Akt1 and Akt2 were not affected but pAkt levels were markedly decreased (approximately 75% lower), as shown by anti-Ser473 and anti-Thr308 Western blotting (Fig. 4a).

To assess whether downregulation of EF1α expression was associated with decreased phosphorylation of a specific Akt isoform, Akt1 and Akt2 were immunoprecipitated by use of isoform-specific antibodies and levels of pAkt assessed by analysis of Ser 473 phosphorylation. As shown in Fig 4b, downregulation of EF1α expression led to a marked decrease of pAkt1 whereas levels of pAkt2 were reduced less.

Thr308 is phosphorylated by PDK1 directly [20], while Ser473 is phosphorylated by TORC2, a complex of four proteins [21]; thus, we began to investigate the role of EF1α in Akt activation by assessing whether it forms a complex with PDK1. Upon transfection in SkBr3 cells, FLAG-tagged PDK1 was in complex with endogenous EF1α and pAkt but not with β-actin (Fig. 4c), raising the possibility that EF1α interacts with PDK1 facilitating Akt\phosphorylation at Thr308.

### Effect of EF1α downregulation on colony formation of breast cancer cells

**Figure 5:**
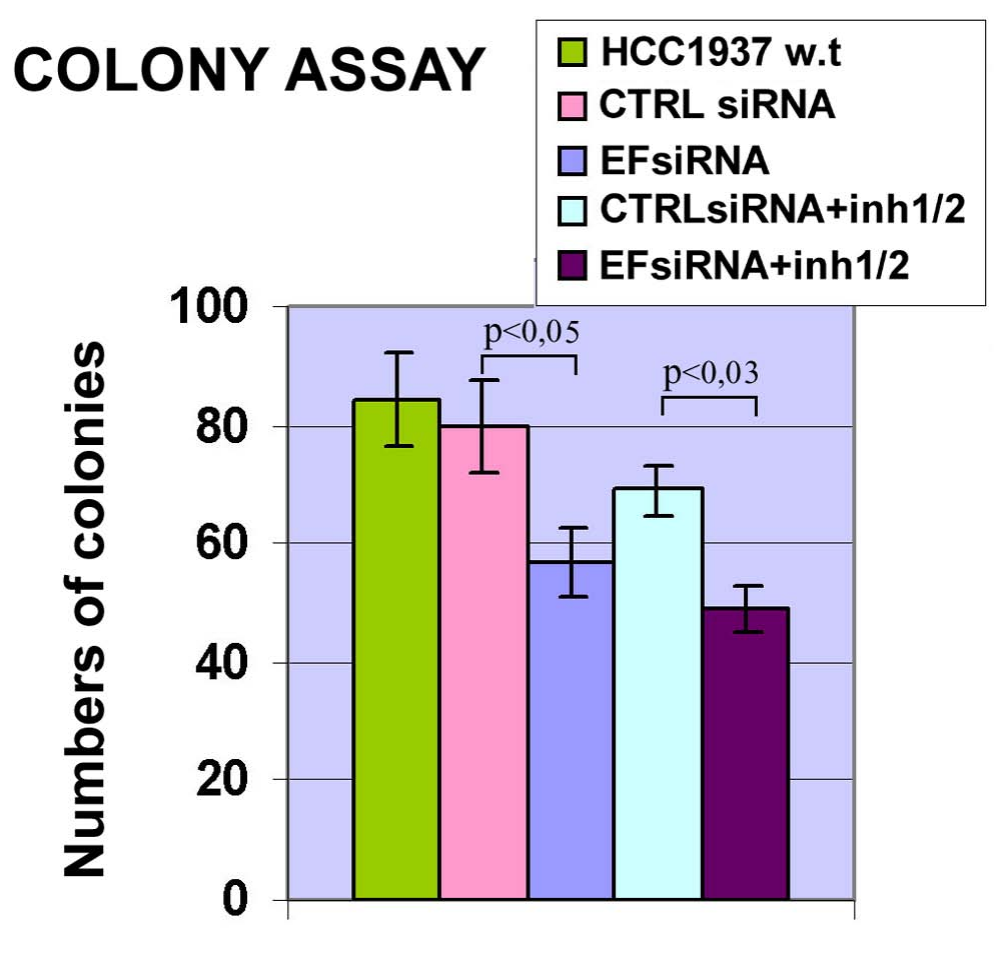
**Colony formation of HCC1937 cells transfected with EF1α siRNA and/or treated with pAkt inhibitors**. HCC1937 cells were transfected with 100 nM of EF1α or CTRLsiRNA and 24 h later counted and plated in 60-mm diameter dishes (3,000 cells for dish). For the co-treatment with siRNAs and the Akt inhibitor, transfected cells were plated in the presence of the Akt inhibitor (final concentration of 7 μM). After 14 days, cells were fixed and stained with crystal violet. Results are representative of three independent experiments with triplicate counts.

To investigate the role of EF1α for breast cancer cell survival, colony formation of HCC1937 cells was assessed after treatment with the EF1α siRNA. 24 h after transfection, 3,000 cells were plated in 60-mm diameter dishes and colonies were counted 14 days later. The number of colonies formed by EF1α siRNA-transfected HCC1937 cells was approximately 30% lower of that from non-transfected parental cells or cells transfected with CTRL siRNA (Fig. 5) and the difference was statistically significant [Student's *t*-test, P < 0.05]. A similar inhibitory effect of the EF1α siRNA (approximately 27%) was also observed in soft agar colony formation assays (see Additional File 4). To determine whether further suppression of pAkt activity could enhance the inhibitory effect of the EF1α siRNA on colony formation, HCC1937 cells were transfected with EF1α siRNA or CTRL siRNA and 24 h later seeded in 60-mm diameter dishes (3,000 cells/dish) in the presence of pAkt inhibitors and assessed in clonogenic assays (Fig. 5). Cells transfected with EF1α siRNA and treated with the Akt1/2 inhibitor formed 38% fewer colonies than cells treated with CTRL siRNA only, 8% fewer colonies than cells treated with the EF1α siRNA and 18% fewer colonies than cells treated with CTRL siRNA and the Akt inhibitors. Downregulation of EF1α expression and pAkt activity also led to reduced colony formation of SkBr3 cells (see Additional File 5).

### Effect of EF1α downregulation on cell cycle activity of HCC1937 cells

To assess whether inhibition of EF1α expression affects the proliferation of HCC1937 cells, we compared the cell cycle activity of CTRL- and EF1α siRNA-transfected HCC1937 cells by DNA content analysis. As shown in Table 1, inhibition of EF1α expression by EF1α RNAi induced a modest increase in the proportion of G1 cells and a correspondent decrease in S phase cells, in comparison to HCC1937 cells transfected with the CTRL siRNA. Compared to CTRL siRNA-transfected cells, a higher fraction of cells transfected with the EF1α siRNA had a subG1 DNA content (1.8% vs 5%) which is indicative of apoptosis.

**Table 1:** Effect of EF1α downregulation and pAkt inhibition on cell cycle distribution of HCC1937 cells

*Cells*	**G0/G1**	**S**	**G2/M**	**Apoptosis**
CTRLsiRNA	60	18.8	20.3	1,8

EFsiRNA	69.4	10.5	17.2	5

CTRLsiRNA + inhib.	63.3	18.8	15.8	4.6

EFsiRNA + inhib.	77.5	10.5	7.6	6.4

Representative of three different experiments with similar results. Values indicate % of cells in different cell cycle phases and apoptosis.

The cell cycle distribution of HCC1937 breast cancer cells was also analysed after EF1α RNAi and pAkt inhibition. Treatment with the Akt inhibitor of EF1α siRNA-transfected cells suppressed the cell cycle more efficiently with a statistically significant increase in the fraction of G1 phase cells (77% in EF1α siRNA-silenced and Akt inhibitor-treated cells vs. 63.3% for cells transfected with the CTRL siRNA and treated with Akt1-1/2 and 69.4% for cells transfected with the EF1α siRNA) and a proportional statistically significant decrease in the number of G2 phase cells (7.6% vs. 15.8% or 17.2%, respectively). The number of apoptotic cells was not affected by the simultaneous EF1α downregulation and treatment with the pAkt inhibitor.

### Effect of EF1α downregulation on invasion of HCC1937 cells

**Figure 6:**
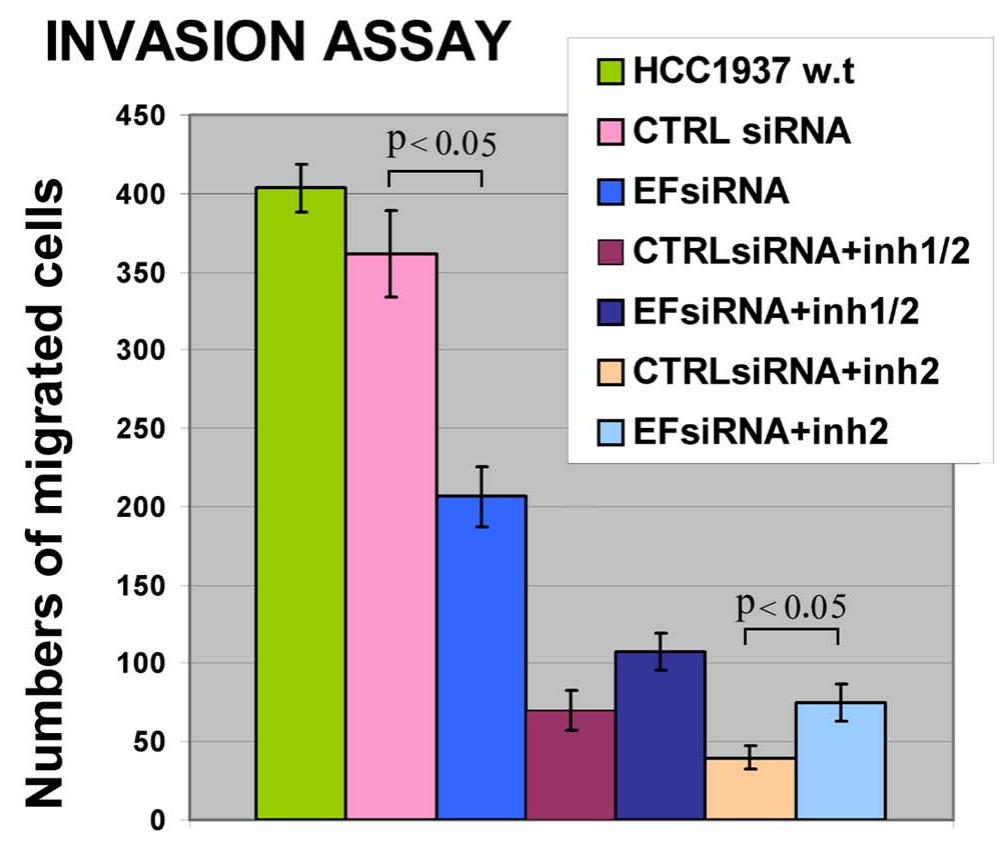
**Invasion of HC1937 cells measured by matrigel assays**. Numbers of migrated cells were counted and each data point represents the mean of three independent experiments.

EF1α binds to actin filaments and microtubules and is involved in actin cytoskeleton remodelling [22,23]. Furthermore, ectopic EF1α2 expression significantly enhanced migration of BT549 breast cancer cells [19]. To assess whether endogenous levels of EF1α were required for breast cancer cell invasion, HCC1937 cells were transfected with the EF1α siRNA to knock-down EF1α expression and invasion tested using Matrigel-coated transwell chambers. Compared to HCC1937 cells transfected with the CTRL siRNA, those transfected with the EF1α siRNA showed approximately a 50% decrease in migration [Student's *t*-test, P < 0.05]; by contrast, invasion of CTRL siRNA-transfected HCC1937 cells was only slightly reduced compared to parental HCC1937 cells, probably reflecting transfection toxicity in this assay (Fig. 6).

Lower pAkt activity in EF1α siRNA-transfected HCC1937 cells might be involved in the reduced invasion of these cells; thus, the number of siRNA-transfected HCC1937 cells migrated trough Matrigel was assessed after treatment with the pAkt2 or the dual pAkt1/pAkt2 inhibitor.

As shown in Fig. 6, simultaneous inhibition of Akt1 and Akt2 activity caused approximately an 80–85% decrease in invasion; interestingly, the pAkt2 inhibitor was slightly more potent than the dual pAkt1/pAkt2 inhibitor in suppressing the invasiveness of HCC1937 breast cancer cells.

Since expression of activated Akt1 blocked the in vitro migration and invasion of three breast cancer cell lines through Matrigel [24], the more potent effect of the specific Akt2 inhibitor in our assays could reflect the anti-migratory activity of residual pAkt1.

## Discussion

The goal of the present study was to identify novel pAkt- interacting proteins and to assess their role in breast cancer cells. To this end, pAkt-interacting proteins were identified by anti-pAkt immunoprecipitation and 2-D electrophoresis followed by in-gel digestion and mass spectrometry analysis of spots exclusively or predominantly present in the pAkt interactome; we reasoned that a protein interacting with pAkt could be a substrate or a modulator of Akt activity. One of the pAkt interacting proteins identified in the proteomic analyses was EF1α. While we were investigating the interaction of EF1α with Akt, Lau *et al*. [25] showed that EF1α co-immunoprecipitates with HA-tagged Akt2, possibly through a complex with β-tubulin. In our mass spectrometry analysis, the presence of EF1α in the pAkt interactome was confirmed in several experiments, but β-tubulin was not detected, suggesting that EF1α and pAkt can also interact independently of β-tubulin.

Since our anti-pAkt antibody does not distinguish between the pAkt isoforms, we used antibodies specific for the Akt 1 or the Akt2 isoform and specific Akt kinase inhibitors to assess the presence of EF1α in the Akt1 and Akt2 interactome of HCC1937 cells.

These analyses revealed that the interaction between Akt2 and EF1α depends on the phosphorylation status of Akt2, the most abundant and active isoform in HCC1937 cells and in breast cancer tumors [26]. A similar phosphorylation-dependent mechanism is also involved in the Akt1-EF1α interaction, since endogenous EF1α was in complex with Myr-Akt1 but not with the phosphorylation-deficient double mutant T308A/S473A Akt1.

Many experiments have demonstrated that Akt-dependent signaling pathways are crucial for normal cell growth and that their deregulation influences cellular responses associated with the cancer phenotype [2,27]

Since abnormal Akt activation is well documented in many human malignancies [7] and EF1α might have oncogenic effects in breast cancer [17,28], the interaction between EF1α and pAkt2 (and/or pAkt1) could represent an important mechanism for the regulation of Akt-dependent signaling pathways in cancer.

Although EF1α associates with pAkt1 and pAkt2 and contains a putative Akt phosphorylation site, our "in vitro" kinase assays indicate that is not an Akt substrate. However, down-regulation of EF1α expression by RNAi led to decreased pAkt expression with an apparently more pronounced effect on the Akt1 isoform, consistent with the reported Akt activation by ectopic EF1α2 expression [19].

The mechanisms involved in the regulation of Akt activity by EF1α are unknown; our finding that EF1α interacts with PDK1 raises the intriguing possibility that it forms a complex with PDK1 and Akt facilitating PDK1 phosphorylation of Akt at Thr 308.

Downregulation of EF1α expression diminished the clonogenic potential of HCC1937 and SkBr3 cells, suggesting that EF1α regulates this aspect of breast cancer cell phenotype; suppression of Akt activity by Akt inhibitors induced only a modest but not statistically significant decrease of colony formation and combining the EF1α siRNA with Akt inhibitors had only a modest additive effect.

DNA content analysis revealed that EF1α downregulation and pAkt1/pAkt2 inhibition suppressed cell proliferation and induced apoptosis.

Since the role of pAkt-substrates in cell cycle progression and apoptosis is well established [2,29,30], the effect of EF1α downregulation on the cycle distribution of HCC1937 cells could be due to decreased Akt activity. However, the effect of EF1α siRNA was more potent than that pharmacological inhibition of pAkt1/pAkt2 and was amplified by simultaneous treatment with the pAkt1/pAkt2 inhibitors.

Together, these data suggest that the effects of EF1α downregulation on proliferation and survival of breast cancer cells are, in part, Akt-independent probably reflecting the role of EF1α in mRNA translation and protein synthesis.

EF1α depletion by RNA-interference also decreased the invasion of HCC1937 cells, suggesting that EF1α might have a role in tumor metastasis. Previous studies have shown a functional relationship between EF1α expression and invasion; in particular, expression of EF1α2 appears to increase cell migration, EF1α mRNA levels correlate with increased metastatic potential in mammary adenocarcinoma and EF1α protein is overexpressed in metastatic compared to non metastatic cells [16,19,31] However, Kulkarni *et al*. [18] reported that EF1α2 expression is associated with good prognosis and suggested that EF1α2 may not be able to activate an effective metastatic program. The modest effect of EF1α downregulation on the metastatic potential of HCC1937 cells is likely to reflect the partial inhibition of pro-metastatic pAkt2 more than loss of anti-metastatic pAkt1 as pAkt2 is the predominant Akt isoform in HCC1937 cells. Consistent with this interpretation, direct inhibition of Akt activity by the Akt inhibitors used here suppressed the invasiveness of HCC1937 cells more efficiently than EF1α downregulation and the specific Akt2 inhibitor was slightly more potent of the dual Akt1/Akt2 inhibitor, a finding in keeping with Akt1 and Akt2 having distinct roles in regulating the motility of breast cancer cells [11,31,32]. The simultaneous EF1α depletion and pAkt inhibition did not reduce further the invasiveness of HCC1937 cells; if anything, the effect of the Akt inhibitors was attenuated by EF1α downregulation, consistent with a more profound inhibition of anti-metastatic pAkt1. However, the effect of active Akt1 on migration and invasion might be cell line and/or cell-type specific [32,33], raising the possibility that downregulation of EF1α might have different effects on the migration of other cancer lines, depending on the relative concentration and activity of Akt isoforms. Since the differences in migration induced by the combination of EF1α siRNA and the Akt inhibitors were modest (only that between CTR siRNA plus Akt2 inhibitor versus EF1α siRNA plus Akt2 inhibitor was statistically significant), it is also possible that they reflect experimental variability.

## Conclusion

In summary, we have shown that EF1α is a pAkt-interacting protein which modulates the activity of pAkt1 and pAkt2 and regulates the proliferation, survival and motility of breast cancer cells. Our results are consistent with previous studies suggesting that EF1α promotes tumorigenesis and indicate that expression of EF1α is required for many properties of breast cancer cells via Akt-dependent and -independent mechanisms. The Akt-dependent effects of EF1α may be, in part, due to the concentration of Akt isoforms, suggesting that investigating the mechanisms responsible for differential Akt isoform expression may be necessary to further understand the role of EF1α in the biology of breast cancer cells.

## Methods

### Plasmids

HA-tagged Myr-Akt1 and T308A/S473A-Akt1 in the pCMV6 vector were the kind gift of Dr. A. Bellacosa (IRE, Roma, Italy); FLAG-tagged PDK1 was the kind gift of Dr H. Ha (Chungbuk National University, KOREA)

### Cell lines and cell cultures

HCC1937 cells were grown in RPMI 1640 medium (Invitrogen-GIBCO, Carlsbad, CA, USA) supplemented with 10% fetal bovine serum (FBS, Gibco), 2 mM glutamine, 100 U/ml penicillin and 100 ug/ml streptomycin.

SKBr3 cells were cultured in Dulbecco's modified Eagle's medium (Gibco) supplemented with 10% FBS (Gibco), 2 mM glutamine, 100 U/ml penicillin and 100 μg/ml streptomycin. Akt inhibitors (pAkt1/2 and pAkt2) (Barnett et al, 2005) were kindly provided by Dr. DeFeo-Jones (Merck Research Laboratories, West Point, PA, USA). The pAkt1/2 and the selective pAkt2 inhibitors were used at a final concentration of 7 μM for 24 h, conditions at which pAkt was essentially undetectable in HCC1937 cells (see Additional File 6). Cells were treated when 60% confluent.

### Immunoprecipitation

For immunoprecipitation of endogenous or ectopic HA-tagged Akt, lysates were precleared with protein G Agarose beads (Oncogene/Calbiochem Laboratories, Cambridge, MA) at 4°C for 45 min and incubated with specific antibodies using conditions suggested by the vendor. To achieve pAkt depletion, saturating anti-pAkt immunoprecipitation was performed for 5 h with an excess of anti-pAkt antibody-conjugated beads. After beads removal, lysates were incubated with the indicated antibody precoated with protein G Agarose beads for 2 h at 4°C. Beads were washed once with lysis buffer and twice with 10 mM Tris-HCL pH 7.4. For IPs, monodimensional electrophoresis and western blot analysis, beads were eluted with 2× Laemmli sample buffer supplemented with 100 μM DTT at 95°C for 7 min.; for 2D-MS analysis, elution was carried out using 2D rehydration buffer (8 M Urea, 2% CHAPS, 50 mM DTT, 30 min/37°C) and appropriate ampholytes (Bio-Rad).

### Protein extraction and western blot

To obtain whole cell lysates, cells were suspended in detergent lysis buffer, disrupted for 20 min at 4°C on a rotary shaker and centrifuged at 13.000 × g for 30 min. The pellet was discarded and protein concentration in the supernatant measured using a Bredford Protein Assay kit (Bio-Rad, Richmond, CA, USA). For western blot analysis, equivalent amounts of proteins and IPs were resolved by SDS-PAGE, transferred to nitrocellulose membranes and blotted. The anti-Ser473-pAkt antibody (#4051, 1:1000 dilution) was from Cell Signaling Technology (Denvers, MA, USA), the anti-EF1α (#sc-12991, 1:800 dilution) antibody was from Santa Cruz Biotechnology (Santa Cruz, CA, USA), as were the anti-Akt1 and Akt-2 antibodies (#sc-5298 and #sc-5270, 1:1000 dilution). The anti EF1α2 antibody was kindly provided by Dr. Abbott (University of Edinburgh, Molecular Medicine Centre, Western General Hospital, Edinburgh). Anti-HA antibody (#MMS-101P, 1:000) was from Covance (Berkeley, CA, USA).

Secondary antibodies were peroxidase-labeled and peroxidase was detected with Enhanced Chemioluminescence Kit (ECL, Amersham Pharmacia Biotech, UK) or Immobilon Western (Millipore Corporation, Bedford, MA, USA).

### 2-Dimensional electrophoresis

Readystrip IPG strip (pH 4–7, 7 cm Bio-Rad) were reydrated in 2D rehydration buffer containing the protein sample. After IEF (Protean IEF cell, Bio-Rad) strips were subjected to reduction and alchylation reactions (15 min in rehydration buffer containing 10 mg/ml of DTT followed by 15 min in rehydration buffer containing 25 mg/ml of iodoacetamide). For second dimension's separation, 7 cm strips were dipped in the SDS running buffer and placed side by side on the top of the same 10% laboratory-made polyacrylamide gel (size 16 cm × 16 cm) to equalize the conditions of electrophoresis and stain. Gels were stained by the Silver Stain method.

### In-gel protein digestion and mass spectrometry

After gel staining, bands or spots of interest were excided from gels with end-removed pipette tip and transferred into a microcentrifuge tube (0.5 mL). Briefly, protein pieces were destained by incubation with 200 μl of 1:1 solution 30 mM potassium hexacyano-ferrate (III) and 100 mM sodium thiosulphate, washed twice with 100 μl of water for 15 min and shrunk with 100% acetonitrile until the gels turned white. Proteins were then reduced adding 50 μl of a DTT solution (10 mM DTT in 50 mM ammonium bicarbonate) and sequentially alkylated using a IAA solution (55 mM IAA in 50 mM ammonium bicarbonate). A volume of 30 μl of trypsin (Promega, Madison, WI) solution (12.5 ng/μl in 25 mM ammonium bicarbonate) was then added, and the gel pieces were incubated at 4°C for 30 min. After digestion, trypsin solution was removed and the samples were incubated at 37° o/n in the same solution without trypsin. Resulting supernatant representing peptide solution were recoverd and concentrated in a vacuum drier (Savant Speed-Vac concentrator).

After resuspensin in 5% formic acid, extracted peptides were sent to C.I.G.S. (Centro Interdipartimentale Grandi Strumenti, Università di Modena, Italy) and identificated by ESI/Q-Tof Mass Spectrometry (Waters-Micromass, Manchester, UK). Peptide identity was determined by searching the Swiss-Prot database.

### Glutathione S-Transferase (GST) Fusion Proteins

To synthesize GST fusion proteins, cDNA segments encoding parts of EF1α protein (amino acids 1 – 137) were amplified by RT-PCR from HCC1937 cells and subcloned into the EcoRI-XhoI-digested pGEX5.1 plasmid. Proteins were expressed in BL21 cells by IPTG induction (1 mM, 3 h), and purified on glutathione-agarose beads (Sigma). GST-proteins were eluted in 50 mM Tris-HCl pH 9.0, 30 mM GSH.

### Peptide synthesis

Synthetic peptide KAERERGITID (EFtide) corresponding to amino acids 64–74 of EF1α and RPRAATF (AKTide) a specific substrate of Akt were synthesized by solid phase peptide synthesis method using an automatized peptide synthesizer (model 431-A, Applied Biosystems, Foster City, CA). Crude peptides were purified by preparative reverse phase HPLC and purity was evaluated by analytical reverse phase HPLC (about 95%). Molecular masses of the peptides were confirmed by mass spectroscopy with direct infusion on a Micromass ZMD-4000 Mass Spectrometer (Waters- Micromass).

### In vitro kinase assay

Phosphorylation reactions were performed by incubating the phosphorylatable protein or peptide substrate in 30 μl of a medium containing 20 mM HEPES (pH 7,5), 10 mM MgCl2, 10 mM MnCl2, 1 mM DTT, 50 μM [γ-33P]ATP (specific activity, 2000 cpm/pmol) and 100 ng of full-length recombinant active Akt1 (specific activity 124 nmol/min/mg) or Akt2 (specific activity 43 nmol/min/mg), expressed in Sf9 cells (from SignalChem, Richmond, BC, Canada) (active Akt1 # A16-10G-05, active Akt2 # A17-10H-05) for the indicated time at 30°C.

The phosphate incorporated into substrates was evaluated either by subjecting samples to SDS/PAGE, staining and autoradiography or using phosphocellulose filters [34]. Values obtained represent the mean of at least three independent experiments.

### RNA Interference

siRNA duplex oligoribonucleotides corresponding to the EF1α gene were designed by Block-iT RNAi Designer (Invitrogen). Best results of EF1α downregulation were obtained by using the following EF1α gene-specific sequences beginning at nt 607 (named "EFsiRNA"): sense 5' Flo-GCGCCUACAUCAAGAAGAUdTdT 3', antisense 5' Flo-AUCUUCUUGAUGUAGGCGCTT 3'.

Transfection of siRNA oligos was carried out with Lipofectamine™ 2000 (Invitrogen). Cells were incubated for 24, 48, 72 and 96 h post-transfection and EF1α expression was detected by western blotting. Fluorescent oligos were used to determine uptake efficiency and set optimal lipofection conditions.

### Colony formation and soft agar assay

24 h after transfection, HCC1937 cells were trypsinized, counted, suspended in 15% conditioned medium and seeded for colony formation assays in 60-mm dishes at 3,000 cells per dish. To test the effect of Akt inhibitors, cells were seeded in medium supplemented with pAkt inhibitors (7 μM in DMSO) in the dark; equal volume of DMSO was used in control experiments. After incubation for 14 days, colonies (>30 cells) were stained with crystal violet and counted. Three different experiments were performed in triplicate.

For soft-agar assays, 5,000 cells were pleated in 60-mm-diameter tissue culture plates containing 0.35% top low-melt agarose-0.5% bottom low-melt agarose. After 2 weeks of incubation at 37°C in 5% CO2 and 95% humidified air, colonies that contained 30 or more cells were counted. Experiments were performed in triplicate.

### Invasion Assay

Cell migration was analyzed with the BioCoat Matrigel Invasion Chambers (BD, Becton-Dickinson, San Jose, CA, USA). Control, siRNA-transfected and Akt inhibitor-treated HCC1937 cells were seeded at 40,000 cells in each upper chamber in RPMI 1640 supplemented with 5% FBS. Lower chambers were filled with 750 μl of 5% conditioned medium of confluent HCC1937 cells containing 10% of FBS as a chemoattractant. Chambers were incubated for 22 h at 37°C in a humidified 5% CO_2 _atmosphere, then cells in the upper chamber were removed. Cells adherent to the lower surface of the membrane were fixed with methanol and stained with crystal violet. Cells migrated to the lower surface of the filter were considered to have invaded through the overlying matrix and were counted.

### Flow Cytometry

Adherent cells were trypsinized, centrifuged at 1000 g for 5 min, fixed in ice-cold 70% ethanol and incubated at -20°C for 2 h. Cells were centrifuged, resuspended in PBS and incubated with RNAse A (200 μg/ml) for 30 min at room temperature; then, cells were incubated with propidium iodide (50 μg/ml) for 30 min at room temperature in the dark. Quantification of sub-2N DNA was determined by flow cytometry using Coulter Epics XL (Beckman Coulter, Fullerton, CA).

## Competing interests

The authors declare that they have no competing interests.

## Authors' contributions

LP performed most experiments and wrote initial draft of the paper. OM planned and carried out all kinase assay. CS participated in the RNA interference assay. OC contributed to kinase assay. LC was involved in drafting the manuscript. ER partecipated in the 2D elecrtophoresis and mass spectrometry analysis. CG contributed to Western blot analyses. SC contributed to sample preparations. SAM performed invasion assays. FC performed colony formation assays. GF participated in the statistical analysis. RB contributed to cell cycle analysis. GR was involved in revising the manuscript critically. MF contributed to various versions including the final version of the manuscript. BC designed experiments and wrote final version of paper. All authors read and approved the final manuscript.

## Supplementary Material

Additional file 1 **Interaction between EF1α and Akt in HCC1937 cells**. These experiments demonstrate that the interaction between EF1α and Akt is not limited to SkBr3 cells. Click here for file

Additional file 2 **In vitro kinase assays**. This experiment shows that EF1α is not an in vitro substrate of p-Akt using SDS-PAGE and autoradiography. Click here for file

Additional file 3 **Downregulation of EF1α expression by EF1α siRNAs in HCC1937 cells**. These experiments show downregulation of EF1α mRNA and protein level by specific EF1α siRNAs. Click here for file

Additional file 4 **Soft agar assay of HCC1937 cells transfected with EF1α or CTRL siRNAs**. This experiment demonstrates that colony formation of HCC1937 cells is suppressed in EF1α-silenced HCC1937 breast cancer cells. Click here for file

Additional file 5 **Colony formation of SkBr3 cells treated with EF1α siRNA and/or pAkt inhibitors**. This experiment demonstrates that inhibition of Akt activity potentiates the colony formation suppressive effect of EF1α RNAi in SkBr3 cells. Click here for file

Additional file 6 **Effect of different concentrations of the pAkt1/2 inhibitor on pAkt expression in HCC1937 cells**. This experiments shows dose-dependent inhibition of Akt phosphorylation (Ser 473) in Akt inhibitor 1/2-treated HCC1937 cells. Click here for file
