# Supplementary material for: Elongation Factor 1 alpha interacts with phospho-Akt in breast cancer cells and regulates their proliferation, survival and motility
Source: Mol Cancer. 2009 Aug 3;8:58. doi: 10.1186/1476-4598-8-58 (PMC2727493; doi:10.1186/1476-4598-8-58)

**Additional File 1.** Interaction between EF1α and Akt in HCC1937 cells.

HCC1937 whole cell lysates and lysates of HCC1937 cells subjected to anti- pAkt (Ser 473) IP in saturating conditions to deplete the active kinase were immunoprecipitated with anti-pAkt or anti-Akt1/Akt2 antibody, respectively. IPs were separated in two equal amounts and subjected to SDS-PAGE and western blotting using anti-Akt1/2 and anti-pAkt antibodies. Western blots were performed using different membranes to distinguish between pAkt and unphosphorylated Akt avoiding superimposition of adjacent bands and background interference. Levels of associated EF1α were also detected in both membranes. Western blots show: (a) pAkt and EF1α  levels in whole cell and p-Akt-depleted lysates. β-Actin was used as loading control; (b) total Akt (Akt1/Akt2) and EF1α in the anti-pAkt IP and in the anti-Akt (Akt1/Akt2) IPs from p-Akt-depleted SkBr3 cell lysate; (c) pAkt and EF1α in the same IP samples as in (b).


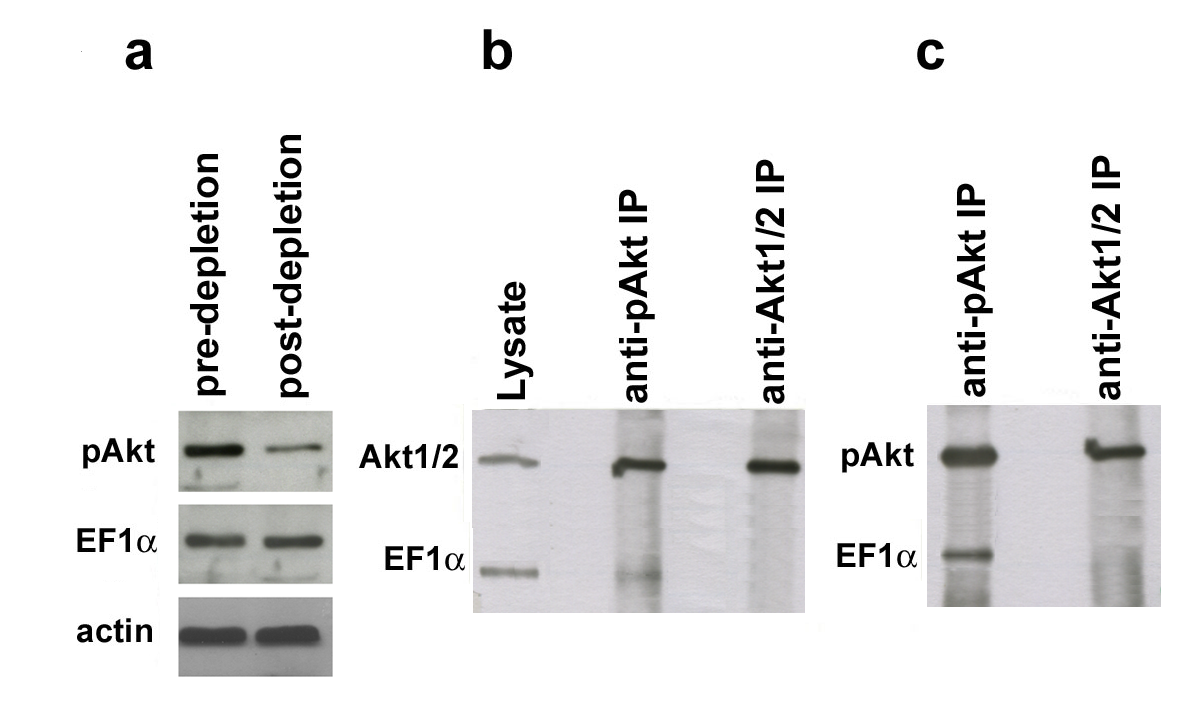

Supplement: Additional file 1 — Interaction between EF1α and Akt in HCC1937 cells. These experiments demonstrate that the interaction between EF1α and Akt is not limited to SkBr3 cells. [file 1476-4598-8-58-S1.doc]
