# Supplementary material for: Elongation Factor 1 alpha interacts with phospho-Akt in breast cancer cells and regulates their proliferation, survival and motility
Source: Mol Cancer. 2009 Aug 3;8:58. doi: 10.1186/1476-4598-8-58 (PMC2727493; doi:10.1186/1476-4598-8-58)

**Additional File 2.** In vitro kinase assays.

GST, GST-EF1α and histone H2B were incubated with recombinant pAkt1 in kinase buffer containing [γ-^32^P]ATP as described in Materials and Methods; samples were analyzed by reducing SDS-PAGE and the gel stained to confirm equal loading of GST fusion protein; dried gel was subjected to autoradiography.


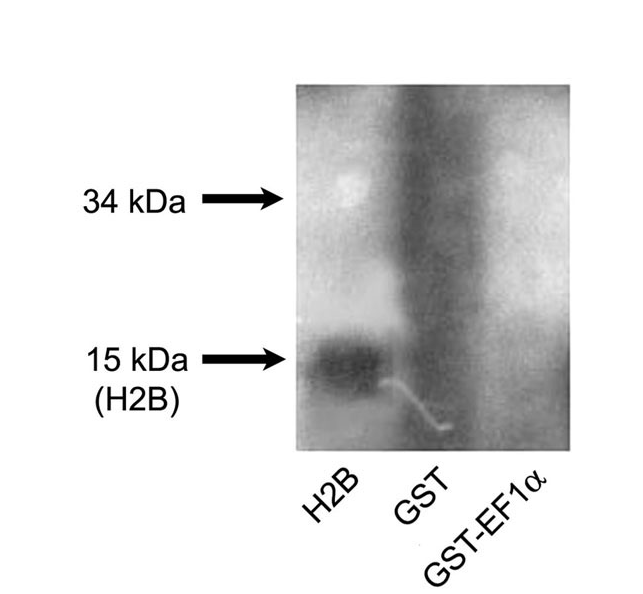

Supplement: Additional file 2 — In vitro kinase assays. This experiment shows that EF1α is not an in vitro substrate of p-Akt using SDS-PAGE and autoradiography. [file 1476-4598-8-58-S2.doc]
