# Supplementary material for: Elongation Factor 1 alpha interacts with phospho-Akt in breast cancer cells and regulates their proliferation, survival and motility
Source: Mol Cancer. 2009 Aug 3;8:58. doi: 10.1186/1476-4598-8-58 (PMC2727493; doi:10.1186/1476-4598-8-58)
Supplement: Additional file 3 — Downregulation of EF1α expression by EF1α siRNAs in HCC1937 cells. These experiments show downregulation of EF1α mRNA and protein level by specific EF1α siRNAs. [file 1476-4598-8-58-S3.doc]

**Additional File 3.** Downregulation of EF1α  expression by EF1α siRNAs in HCC1937 cells.

(a) Time course of EF1α mRNA downregulation. Total RNA was isolated at the indicated times after transfection with EF1α-specific siRNA (indicated 237, 607 and 1108 downstream from the translation initiation codon) and mismatch CTRLsiRNA; Reverse transcription-PCR was performed to detect levels of EF1α and β-actin.

(b) EF1α protein levels in EFsiRNA-transfected cells. Total cell lysates were collected 24, 48 and 96 h after transfection with 100 nM EFsi RNA (607). EF1α was detected with an antibody targeting an epitope common to the 1α1 and 1α2 isoforms. β-actin served as a control for loading normalization. By western blot analysis, EF1α protein levels were significantly reduced in HCC1937 cells transfected with the EFsiRNA but not in cells transfected with the CTRLsiRNA.

Indeed, levels of EF1α protein showed a 72% decrease at 24 h, a 49% decrease at 48 h and returned at the levels of non-transfected HCC1937 cells 4 days post-transfection.


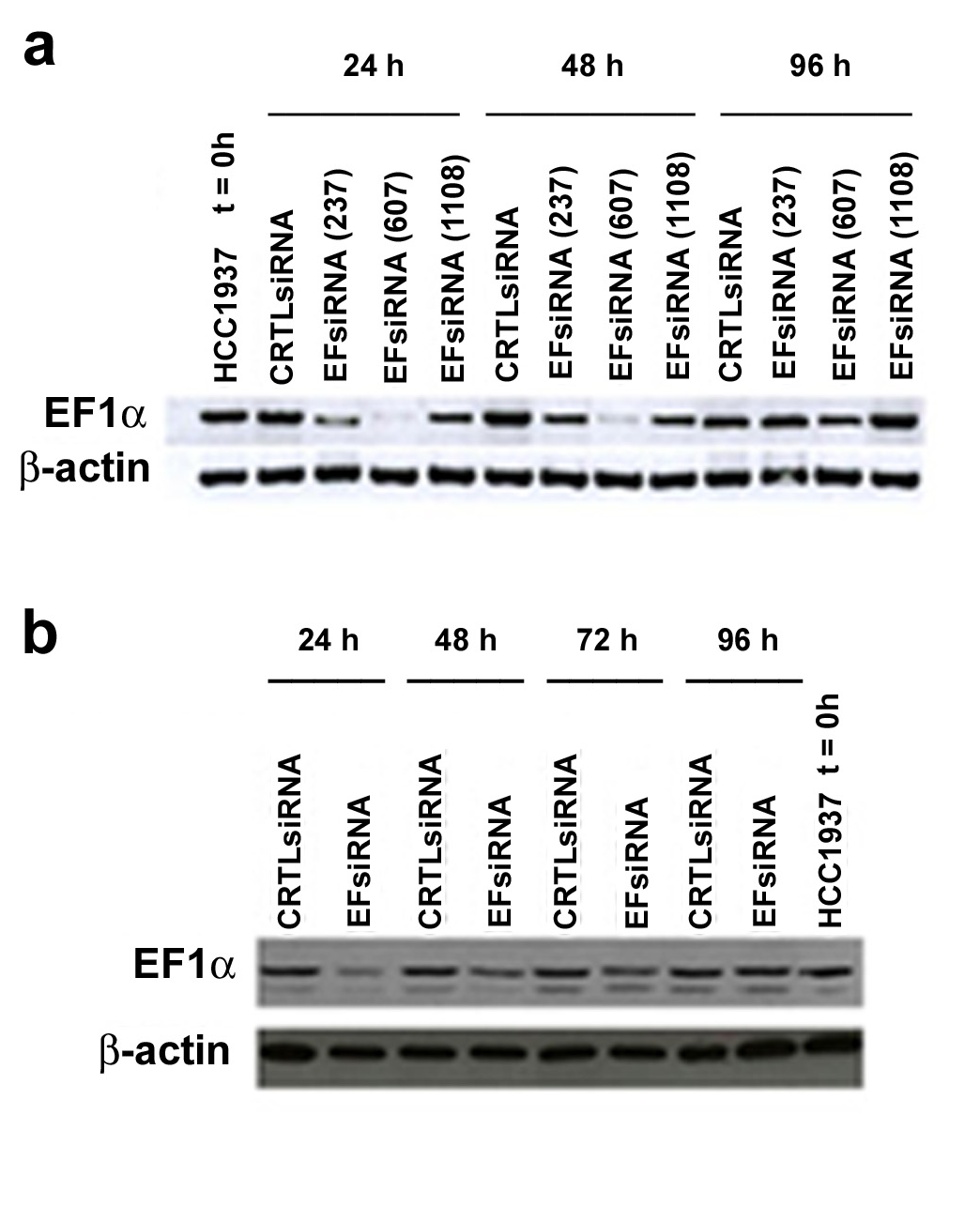


**Methods:**

For high efficient EF1α RNA interference, siRNA duplex oligoribonucleotides corresponding to the EF1α gene were designed by Block-iT RNAi Designer (Invitrogen). The sequences and their complements were Blast-searched against the human genome to ensure that only EF1α was targeted. Oligoribonucleotides were chemically- synthesized and annealed by Invitrogen. Three sequences corresponding to different regions (indicated 237, 607 and 1108 from the start codon) of the coding sequence were chosen to target EF1α (both 1α1 and 1α2 isoforms) and one additional siRNA sequence (without homology to human genes) was used as negative control. Transfection of siRNA oligos was carried out with Lipofectamine^TM^2000 (Invitrogen) in T25 flask (final volume 5 ml) as recommended by the manufacturer. Briefly, the day before transfection, cells were trypsinized, counted and seeded without antibiotics at 6 x 10^5^ cells / T25 flask, so that they were 50% confluent on the day of transfection. Lipofectamine^TM^2000 was diluted in RPMI (18 μl of Lipofectamine to 500 μl of RPMI) and supplemented to the dsRNA oligo (100 nM); the formulation was left for 25 min at room temperature and then added to the cells drop by drop. Cells were incubated for 24, 48, 72 and 96 h and EF1α expression was detected by western blotting. Fluorescent dsRNA oligos were used to determine uptake efficiency and set optimal transfection conditions.
