# Supplementary material for: Elongation Factor 1 alpha interacts with phospho-Akt in breast cancer cells and regulates their proliferation, survival and motility
Source: Mol Cancer. 2009 Aug 3;8:58. doi: 10.1186/1476-4598-8-58 (PMC2727493; doi:10.1186/1476-4598-8-58)
Supplement: Additional file 4 — Soft agar assay of HCC1937 cells transfected with EF1α or CTRL siRNAs. This experiment demonstrates that colony formation of HCC1937 cells is suppressed in EF1α-silenced HCC1937 breast cancer cells. [file 1476-4598-8-58-S4.doc]

**Additional File 4.** Soft agar assay of HCC1937 cells transfected with EF1α and CTRL siRNAs.

14 days after plating, colonies of at least 30 cells were counted. Results are representative of three independent experiments with triplicate counts. P-value<0.05

**Methods:**

For soft-agar assays, cells were plated at a density of 5,000 in 60-mm-diameter tissue culture plates containing 0.35% top low-melt agarose–0.5% bottom low-melt agarose. After 2 weeks of incubation at 37° C in 5% CO2 and 95% humidified air, colonies consisting of 30 or more cells were counted. Experiments were performed in triplicate.
