# Supplementary material for: Elongation Factor 1 alpha interacts with phospho-Akt in breast cancer cells and regulates their proliferation, survival and motility
Source: Mol Cancer. 2009 Aug 3;8:58. doi: 10.1186/1476-4598-8-58 (PMC2727493; doi:10.1186/1476-4598-8-58)
Supplement: Additional file 5 — Colony formation of SkBr3 cells treated with EF1α siRNA and/or pAkt inhibitors. This experiment demonstrates that inhibition of Akt activity potentiates the colony formation suppressive effect of EF1α RNAi in SkBr3 cells. [file 1476-4598-8-58-S5.doc]

**Additional File 5.** Colony formation of SkBr3 cells treated with EF1α siRNA and/or pAkt inhibitors.

SkBr3 cells were treated with the Akt inhibitor or transfected with 100 nM of EFsiRNA or CTRLsiRNA. 24 h after treatment, cells were counted and plated in 60-mm diameter dishes (3,000 cells for dish). For the co-treatment with siRNAs and Akt inhibitor, transfected cells were plated in the presence of the Akt inhibitor added to the medium at a final concentration of 7 uM. After 14 days, cells were fixed and stained with crystal violet. Results are representative of three independent experiments with triplicate counts.
