# Supplementary material for: Elongation Factor 1 alpha interacts with phospho-Akt in breast cancer cells and regulates their proliferation, survival and motility
Source: Mol Cancer. 2009 Aug 3;8:58. doi: 10.1186/1476-4598-8-58 (PMC2727493; doi:10.1186/1476-4598-8-58)
Supplement: Additional file 6 — Effect of different concentrations of the pAkt1/2 inhibitor on pAkt expression in HCC1937 cells. This experiments shows dose-dependent inhibition of Akt phosphorylation (Ser 473) in Akt inhibitor 1/2-treated HCC1937 cells. [file 1476-4598-8-58-S6.doc]

**Additional File 6.** Effect of different concentrations of the pAkt1/2 inhibitor on pAkt expression in HCC1937 cells.


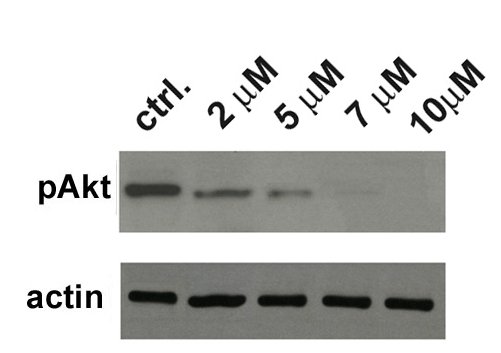


**Methods:**

The pAkt1/2 inhibitor was tested at final concentration of 2μM, 5μM, 7μM and 10μM for 24h. Cells were treated when 60% confluent. Optimal pAkt down-regulation without short-term toxic effects on cell proliferation was obtained at a concentration of 7μM.
